# Toward a P300 Based Brain-Computer Interface for Aphasia Rehabilitation after Stroke: Presentation of Theoretical Considerations and a Pilot Feasibility Study

**DOI:** 10.3389/fnhum.2016.00547

**Published:** 2016-11-11

**Authors:** Sonja C. Kleih, Lea Gottschalt, Eva Teichlein, Franz X. Weilbach

**Affiliations:** ^1^Institute of Psychology, University of WürzburgWürzburg, Germany; ^2^Department of Neurology, Klinik Bavaria Bad KissingenBad Kissingen, Germany

**Keywords:** brain-computer interface (BCI), aphasia, Broca, stroke rehabilitation, P300 speller, training, user-centered design

## Abstract

People with post-stroke motor aphasia know what they would like to say but cannot express it through motor pathways due to disruption of cortical circuits. We present a theoretical background for our hypothesized connection between attention and aphasia rehabilitation and suggest why in this context, Brain-Computer Interface (BCI) use might be beneficial for patients diagnosed with aphasia. Not only could BCI technology provide a communication tool, it might support neuronal plasticity by activating language circuits and thereby boost aphasia recovery. However, stroke may lead to heterogeneous symptoms that might hinder BCI use, which is why the feasibility of this approach needs to be investigated first. In this pilot study, we included five participants diagnosed with post-stroke aphasia. Four participants were initially unable to use the visual P300 speller paradigm. By adjusting the paradigm to their needs, participants could successfully learn to use the speller for communication with accuracies up to 100%. We describe necessary adjustments to the paradigm and present future steps to investigate further this approach.

## Introduction

Brain-computer interfacing (BCI) does not require motor control, but instead either willful brain activation or attention allocation to certain stimuli (Wolpaw and Wolpaw, 2012). Successful BCI use was reported in severely motor-impaired but cognitively intact patients (Hoffmann et al., 2008; Silvoni et al., 2009; Nijboer et al., 2010). One possible BCI input signal is the P300 which represents a positive deflection in the EEG occurring 300 ms after the onset of a relevant stimulus, or target, presented within a stream of irrelevant stimuli, or non-targets (oddball paradigm, Sutton et al., 1965). In a classic visual P300 spelling paradigm (Farwell and Donchin, 1988), letters of the alphabet are arranged in a matrix of rows and columns. The target stimulus is the letter to be selected which is highlighted (flashed) once in the row and once in the column accordingly. The non-target stimuli are to be ignored by the BCI user. The BCI detects the P300 response to the target stimulus cell, displays the target letter on a computer screen and thereby allows for communication (Farwell and Donchin, 1988).

Even though establishing communication in paralyzed patients has been one of the major goals of BCI research for decades, BCIs were most recently also used in rehabilitation contexts (Daly and Huggins, 2015) including rehabilitation after stroke. Morone et al. (2015) reported on the use of BCI technology for rehabilitation of the upper limb by having patients undergo a BCI based training schedule. Patients had to imagine movements with their paralyzed arms and hands and these imagined movements were translated into movements of a virtual hand. The authors hypothesized this training to boost neuronal plasticity after stroke and thereby support motor rehabilitation. And indeed, patients improved their ability to move hands and arms (as measured by the Fugl-Meyr scale) to a clinically relevant extent.

Besides possible motor impairments, manifold cognitive impairments might occur after stroke (Jokinen et al., 2015). Up to 50% of all stroke survivors are affected by attention impairments (Leśniak et al., 2008) and up to 30% suffer from language production or language comprehension deficits (= aphasia, Flowers et al., 2013). In the pilot study presented here, we aimed at focusing on aphasia caused by lesions in the opercular and triangular part of the inferior frontal gyrus, the Broca area, the temporoparietal region and related circuits of the brain (motor aphasia e.g., Berndt and Caramazza, 1999). Usually, afferent fibers receive information from the primary and secondary auditory cortices and several association fields. Efferent fibers to the precentral gyrus are activated directly via the basal ganglia and indirectly via the thalamus and cerebellum (Trepel, 2011). Corticonuclear fibers activate nuclei in the brainstem, which initiate muscle activation in the larynx and the pharynx. Mimics and language are produced. With motor aphasia, language can still be perceived and understood, but language production is limited or impossible. In case language is not completely lost, sentences are short, word production is flawed and self-expression is very demanding (Kelly et al., 2010).

Language therapy as provided by the healthcare system was shown to have positive effects mainly in early rehabilitation phases (Robey, 1994) and for patients who suffered from language comprehension disorders (Kelly et al., 2010). Therefore, traditional language therapy is highly valuable, but unfortunately might not be sufficient. Half of the aphasic patients do not recover fully (Hartje and Poeck, 2000). In the long term, not only might the psychological burden become manifest as a consequence of chronic aphasia (Gainotti, 1997), but also the economic situation might deteriorate (Hinckley, 1998) as in many workplaces employees must be able to communicate elaborately. Therefore, aphasia rehabilitation after stroke is a challenge deserving more attention by research and rehabilitation care.

BCI use for communication was tested and reported successful in eight participants diagnosed with aphasia (Shih et al., 2014). The authors used a visual P300 based checkerboard paradigm and reported achieved accuracies of between 60% and 65%. Another approach was presented most recently by Musso et al. (2016) who presented an auditory BCI system being used by a patient with chronic aphasia. Their goal was to identify neuronal markers of auditory attention. However, in both mentioned studies, the theoretical background for BCI use and therefore, attention allocation which might be useful in aphasia rehabilitation, were missing. To our knowledge, the presented relation between attention, aphasia and BCI use and its potential implications for aphasia rehabilitation are the first theory-based considerations on this research topic.

### Aphasia and Attention

Hula and McNeil (2008) suggested aphasia to result from an attention deficit, potentially caused by a lesion which disrupts regular brain functioning. They hypothesized a completely functional brain to be capable of simultaneous processing, which in language processing would translate to understanding the dialog partner, thinking about one’s own input and producing language. Of course, also additional neuronal circuits responsible for other cognitive abilities such as memory, might be triggered together with the motor system to finally contribute to the conversation. However, in aphasia, simultaneous processing was assumed to be impossible due to lesions caused by brain injury or stroke. As attention, language processing was hypothesized to be based on widespread neuronal activation throughout the cortex. Therefore, disruption of these circuits’ interactions might cause aphasic symptoms.

One reason why aphasia was believed to be related to attention deficits was the observation that language production could be stimulated in participants with aphasia (Murray, 1999). If language deficits followed the rules of purely linguistic models, such stimulation of language production should be impossible. Also the fact that language performance of patients with aphasia could be improved by manipulation of stimulus presentation (e.g., loudness or presence/absence of background noise) did not fit in a purely linguistic model of aphasia (Murray, 1999). In a study with participants diagnosed with aphasia, with right hemispheric brain damage or no brain damage, it was shown that for the single task condition, word retrieval performances did not differ between groups. In the dual-task condition, participants with aphasia and the ones with the right hemispheric lesion performed significantly worse as compared to participants without brain lesion. Therefore, it was concluded that attention resources play a major role in language processing (Murray, 2000). Also in another study, no performance differences were found between participants with aphasia and controls in an easy tone discrimination task. However, when participants entered the dual-task condition, (sorting cards while discriminating the tones), participants with aphasia performed significantly worse as compared to controls (Erickson et al., 1996). These results suggest a brain anatomical overlap between the occurrence of aphasic symptoms and attention deficits. In aphasia, middle and inferior temporal cortices, the temporoparital cortex, inferior parietal and premotor cortices, the cerebellum, the supplementary motor area, the anterior cingulate gyrus and the thalamus might all be affected (Price, 1998). For attentional control, the fronto-parietal network (Shomstein et al., 2012) and thalamus (Trepel, 2011) were suggested to play a major role. More precisely, the intraparietal sulcus, the inferior parietal lobe and the dorsal premotor cortex were stated to be parts of the fronto-parietal attention network (Ptak, 2012). Thus, it seems comprehensible that brain lesions caused by stroke might lead to attention deficits and at the same time to aphasia symptoms. Furthermore, attention deficits might hinder language processing in patients with aphasia. Murray (1999) summarized her review with the notion that there seems sufficient evidence to believe in a role of attention in aphasia as reduction of task demands increased task performance in patients with aphasia. She recommended to decrease complexity of tasks to support aphasic patients’ language skills.

### Aphasia and P300

Changes in P300 characteristics following aphasia were reported for example by Peach et al. (1992) who found increased latencies in participants with aphasia as compared to healthy controls in an auditory oddball paradigm. Decreased P300 amplitudes were reported for patients diagnosed with left-hemispheric lesion and aphasia as compared to controls in a lexical semantic processing task (Hagoort et al., 1996). Nolfe et al. (2006) found the P300 to be predictive of late recovery from aphasia. They judged the presence of the P300 amplitude to be indicative of preserved elementary cognitive function after stroke affecting areas that are relevant for language processing and production. In line with these observations, the medial temporal lobe and integrity of the temporoparietal junction were suggested to play a role in modulation or even creation of the P300 amplitude (Picton, 1992; Knight and Scabini, 1998; Polich, 2007). Lesions in these areas however, still allowed for detection of a P300 amplitude (Knight and Scabini, 1998) even if the amplitude was found to be decreased. Thus, the assumption of network structures being involved in P300 origin and modulation seems most acceptable (Picton, 1992; Polich, 2007).

### P300 BCI Use in Aphasia

Implementing visual P300 BCI could potentially tackle two goals, both contributing to aphasia rehabilitation: first the P300 amplitude is dependent on attention allocation and therefore an indicator of attention allocated to the task (Johnson, 1986; Polich, 2007). So if BCI training is usable as an attention training because letters have to be selected by focusing attention, we should see an increased P300 amplitude on the psychophysiological level. However, this assumption cannot be transferred to people diagnosed with post-stroke aphasia without investigation as their brains might react differently as compared to brains unaffected by stroke. Increased P300 amplitudes as a result of training to focus on the target stimulus were shown for non-visual BCI paradigms (Baykara et al., 2016; Herweg et al., 2016). Long-term stable P300 amplitudes in a visual speller paradigm were reported for severely motor-impaired participants (Nijboer et al., 2008) even though the P300 might habituate (Ravden and Polich, 1998). We thus need to take the attentional state into account. Second, language that is thought by a patient, therefore is existent in the brain and might be transferred to the environment by BCI use. The patient may not be able to say the word, but could express it via BCI. Thus, neuronal networks producing language might be supported in plasticity, which would facilitate rehabilitation. This might boost neuronal plasticity more efficiently as compared to having the patient write his or her thought, type them or express them otherwise via substitutions.

As the communication goal with the environment is natural speech, it might be argued that using a BCI might strengthen activation of a “non-natural” language output. Therefore, a patient using P300 BCI should be encouraged to attempt pronunciation of BCI spelled words even if language production might currently be impossible. Furthermore, it is known that stroke patients usually do not suffer from one specific deficit to be addressed but that instead, patients present with diverse post-stroke symptoms, such as apraxia, paralysis and impaired vision amongst others (e.g., Jokinen et al., 2015). These symptoms might hinder BCI use or even prevent it completely. Thus, our goal was to investigate whether participants diagnosed with aphasia or even fulfilling exclusion criteria for BCI use, can be trained to use a visual P300 speller and if yes, how.

We hypothesize stroke patients with aphasia to be able to use a P300 based BCI system even if they would usually be excluded from participation in a BCI study. Additionally, we hypothesize the spelling system to be adaptable to the special needs of aphasic patients which might lead to a changed P300 speller design. We hypothesize the ability to focus (attention) as measured neuropsychologically to predict BCI performance in participants diagnosed with post-stroke aphasia. We finally hypothesize the P300 amplitude to increase with training.

## Materials and Methods

### Participants

We included *N* = 5 stroke patients with aphasia. One additional patient was unable to give informed consent to the study, he had to be excluded from participation. All participants received rehabilitation treatment at the Bavaria Clinic in Bad Kissingen, Germany and were also recruited there. The sample consisted of two males and three females (see Table 1). One female participant only participated for one BCI session (E). Throughout her stay in the rehabilitation clinic, she was diagnosed with an acute depressive episode and therefore was unable to participate in further BCI sessions. Participants A to D were right-handed, participant E was left-handed. All participants were selected by medical doctors according to three major inclusion criteria: (1) aphasia (predominantly motor); (2) being able to concentrate for at least half an hour; and (3) being able to understand instructions on how to use a BCI. All participants gave informed consent prior to study participation. This study was approved by the Ethical Review Board of the Psychological Institute at the Julius-Maximilians-University of Würzburg.

**Table 1:** **Participant description, time since stroke event and number of Brain-Computer Interfaces (BCI) training sessions (N°) for each participant**.

Participant	Age	Sex	Diagnoses	Time since stroke event	N° BCI sessions
A	46	M	Intracerebral hemorrhage in left cerebrum (I 61), aphasia, bilateral hemianopsia	1 year	6
B	51	F	Left-sided boundary zone infarction in the area of the middle cerebral artery (I 63), alexia, agraphia, right sided neglect, hemianopsia	1 year	3
C	60	M	Ischemic infarction in the right cerebellar peduncle (I 63), residual dysarthria, aphasia and spastic hemiparesis, 2015: contusion hemorrhage caused by brain injury in the frontobasal and temporobasal area, left-sided	3 years	3
D	83	F	Left-sided media infarction (I 63), aphasia, apraxia	2 months	3
E	53	F	Left-sided hemorrhage in the thalamus and ventricle irruption (I 61)	4 months	1

### Aphasia Severity

Aphasia was diagnosed by professional speech therapists using the Bielefelder Aphasie Screening (BIAS; Richter et al., 2006). The BIAS can be administered in the acute phase within 20–40 min depending on aphasia severity. Aphasia is judged based on the performance in the six subscales: *spontaneous speech, auditory comprehension, automatic language use, semantic lexical performance, reading comprehension and writing of words*. Psychometric properties of objectivity and validity are satisfying. Reliability indices (Cronbach’s alpha) of between 0.79 and 0.97 for all subscales are rather high (Richter et al., 2006). Participants’ percentile rank (PR) and aphasia severity as judged by professional speech therapists were summarized in Table 2.

**Table 2:** **Bielefelder Aphasie Screening (BIAS) test results as percentile rank (PR) and judgment of Aphasia severity**.

Participant	BIAS PR	Aphasia severity	Major deficit
A	29	Medium to severe	Word production and phrasing
B	25	Medium to severe	Phrasing and reading
C	13	Severe	Word production
D	16	Severe	Word production and comprehension
E	40	Mild to medium	Phrasing

### Rehabilitation Treatment

The standard rehabilitation treatment for the participants included here consisted of speech therapy (5 times a week, 30 min a day), neuropsychological training, occupational therapy and physiotherapy. All participants received group therapy programs as well as individualized therapies in all mentioned disciplines, Monday through Friday between 8 am and 5 pm. Our BCI sessions were accommodated in available vacant time slots. All participants received standard rehabilitation treatment for at least 2 weeks before being included in the study (participants B, C, E). Participant A had been admitted for 4 months before entering the study. Participant D had been treated for 4 weeks before entering the study. Two participants (A and B) received computer based attention training (neuropsychological treatment) presented by the RehaCom Software (HasoMed GmbH, Magdeburg, Germany). This training included 30 min of training at least three times a week. Participant A additionally received computer skills training including tasks such as opening documents and internet pages. He received this training every day for at least 30 min per session.

### Attention Tests

To assess attention, we used the attention performance test (german: Testbatterie zur Aufmerksamkeitsprüfung = TAP), administered by the TAP Software package 2.3, (Zimmermann and Fimm, 2012) on a laptop (Lenovo Thinkpad T540, i3 processor, 15.6′′ display). The two square shaped push-button response keys were provided in the TAP2.3 software package. In all tests, reaction time, errors and omissions were measured and evaluated (provided norms) by the software. Results were presented in raw values (RV: number of errors, omissions and reaction times) and as PR (Zimmermann and Fimm, 2012). In the subtest *alertness*, participants had to press the response key as fast as possible after noticing the appearance of a target stimulus cross (x) presented in the center of the screen. This subtest consisted of four runs (22 response targets each) with the first and the last run measuring the reaction time to the target cross stimulus. In runs two and three, the participant was alerted by an auditory warning stimulus presented prior to the target stimulus. Administration time for this test was 12 min and 30 s. The subtest *divided attention* included simultaneous reactions to a visual and an auditory task. In the visual task, crosses switched positions on a pre-defined grid and each time four crosses formed a square (17 times), the response key had to be pressed. At the same time, a high-pitched and a low-pitched tone were alternatingly presented. Each time one of the tones was presented twice in sequence and thereby violated presentation rules (16 times), a response was required. Administration time was 3 min. The *Go-NoGo* test measured selective attention. Two different stimuli were presented, a cross sign (×, 20 targets) and a plus sign (+, 20 targets). A response was only requested for the cross sign but had to be inhibited for the plus sign. Administration time was 2 min. In the *flexibility* subtest, focused attention was investigated. A pair of one letter and one number was presented on the screen (100 pairs in total). One of the two stimuli (letter or number) was presented on the right and the other one on the left of the screen. There were two response keys. The left one (positioned at patient’s left hand) had to be pressed when the target stimulus was presented on the left, the right one (positioned at patient’s right hand) in case the target stimulus was presented on the right. While the target stimulus presentation was following a strict alternating scheme (letter, number, letter…), location for presentation could but did not have to alternate (set-shifting task). Administration time was 5 min. The total TAP administration time was 14 min and 30 s, however, depending on how much instruction the participant needed prior to each subtest, up to 35 min were required for data acquisition. The TAP screen was positioned at a distance of 55–60 cm from the participant.

### BCI Spelling Paradigm and Procedure

Measurements were integrated in the rehabilitation plan of patients by the clinic administration and took place in a room provided by the Bavaria Clinic Bad Kissingen. Measurement appointments varied according to the rehabilitation schedule as we tried to interfere as little as possible with the therapies a participant received. The first BCI session lasted 2 h as prior to the BCI session, we administered the TAP data assessment. All the following BCI sessions lasted one and a half hours and were scheduled within 7 (participants B, C, D) to 12 working days (participant A).

We used a 6 × 6 spelling matrix containing the letters of the alphabet from A to Z and the numerals 0 to 9. For the display we used a Fujitsu screen (L22T, 21.5′′). The matrix cells were 2.5 cm × 2.5 cm in size. Participants were seated approximately 80 cm away from the screen. The BCI paradigm was controlled by the BCI2000 Software (BCI2000; Schalk et al., 2004) and calibrated using the words BRAIN and POWER. We used 10 sequences, therefore each target letter cell was highlighted 20 times (1 trial), 10 times in the row and 10 times in the column. Flash duration was 62.5 ms with an ISI of 250 ms. Subsequent to spelling of a target letter, the screen stayed static for 1000 ms to allow for finding the next letter to spell on the matrix. We planned to assess three copy-spelling runs per session using the words RADIO (radio), BLUME (flower) and FUCHS (fox) which equals 15 letters per session. Depending on the participant’s condition, we added one more word or assessed less words as compared to the planned number (see “Results” Section). After copy-spelling, the goal for patients was to copy-spell their own words. Finally, patients should be able to use the free-spelling mode such that they spelled without the experimenter knowing the target word. Participants were instructed to free-spell words that contained at least three letters. We continuously asked participants whether they needed assistance. The number of free-spelled letters varied across subjects.

### Custom-Made Post Measurement Questionnaire

We asked participants to rate subjective workload concerning the BCI task on a visual analog scale ranging from 0 (= not exhausting at all) to 10 (= very exhausting). We also asked about possible strategies to elicit a P300 (such as counting the target letter). Additionally, the matrix had to be judged concerning the size and arrangement of letters. We were interested in usability and a possible need for additional assistance (by the experimenter or other). We further investigated the benefit of provided assistance and which aspects might be improved.

### Data Acquisition

The P300 speller was controlled by BCI 2000 (Schalk et al., 2004). EEG was measured with an electrode cap (easy cap) with 12 Ag/AgCl electrodes located at positions Fz, FCz, C3, Cz, C4, CPz, P3, Pz, P4, PO7, Oz, PO8 as recommended by Krusienski et al. (2008), referenced to the right and grounded to the left mastoid. Data were filtered online with a high pass of 0.1 Hz, a low pass of 30 Hz and a notch filter of 48–52 Hz. The EEG signal was amplified with a g.USBamp (Guger Technologies, Austria). Impedance was kept below 5 kΩ and sampling rate was 256 Hz. For data classification, online and offline stepwise linear discriminant analysis (SWLDA) was applied (for details see Krusienski et al., 2008).

### Data Analysis

EEG data were corrected for artifacts (>70 μV) and baseline (−100 ms to 0 ms) using Brain Vision Analyzer (BrainProducts, Gilching, Germany). For participant A, 8.22% of the data were excluded due to artifact correction and for participant C, 0.90% of the data. From all other participants we could use all assessed data for further analysis. The P300 was defined as the maximum positive peak between 250 ms and 600 ms after stimulus onset identified by semiautomatic global peak detection on electrode locations Fz, Cz and Pz. Targets and non-targets each were averaged and contrasted for the performed sessions. For classifier training (offline), we used data from the calibration runs from all electrodes. This classifier was used for the online spelling sessions. Every participant underwent one calibration per session with the exception of participant C who underwent two calibration sessions in session 3. To control for possible effects of visual evoked potentials, we re-trained the classifier offline excluding occipital electrodes. Accuracies reached including all electrodes and including the selected electrode subset were reported in the “Results” Section. Dependent variables were: Ability to use the P300 speller, spelling performance as measured in percent of correctly spelled characters, P300 amplitude as an indicator of attention and changes needed in the spelling paradigm to improve usability in the future. PR in the TAP assessment were reported with the average range being indicated by values between 16 and 85. Due to the fact that we can only present data of *N* = 5 participants, no statistical analysis was performed and we report results qualitatively.

## Results

### Usability and Adaptation

We hypothesized patients disgnosed with aphasia to be able to use a P300 speller successfully, however, we also assumed adjustments to existing protocols to be necessary. This hypothesis was confirmed by all participants.

**Figure 1:**
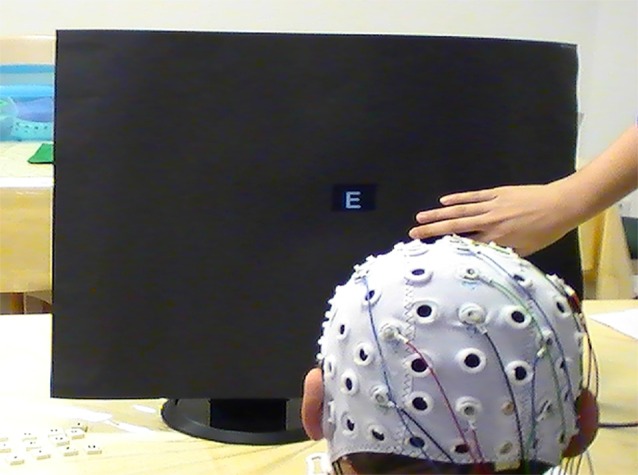
**Cardboard cover assistance held by experimenter’s right hand**.

In participant A, we conducted six sessions in total as after the third session, he could not yet use the free-spelling mode which was a goal to be reached in every patient. In the beginning, this patient also could not use the matrix successfully in copy-spelling mode as he reported major problems in ignoring non-target stimuli. The classifier could not be trained based on the data produced by the participant, even though he reported having understood the task. Therefore, we supported focusing on the target letter by covering the matrix beginning in the second session with a black piece of cardboard on which a square for the presentation of the target letter was spared (see Figure 1). Thus, when using the cardboard, the user could not freely choose the letter to be spelled but was forced to choose the pre-selected (spared) letter. This aid was used also in patients C, D and E, mostly during the first session. All participants were first tested with the standard paradigm in which they were not able to produce classifiable EEG responses during calibration. Using the cardboard changed the usual BCI paradigm such that a target stimulus was classified against background EEG as compared to classifying target vs. non-target stimuli. Still, the BCI user needed to focus attention on the target stimulus as otherwise a random letter would be selected. In their last session, all participants could use the free-spelling mode without any additional devices or assistance.

Patient B reported to suffer predominantly from her alexia as compared to her aphasia symptoms. We tested the effect of word spelling using scrabble letter tiles to compare this training with BCI based spelling. In the letter tile condition prior to the BCI based spelling, the participant had to choose the letter tiles necessary to spell the word and then to arrange the tiles such that they formed the correct word. The patient could read the nine letter word TABELLEN (tables) only after BCI based spelling compared to scrabble tile based spelling. This effect was not replicated for other words which were read successfully immediately after reading the word TABELLEN successfully (APRIKOSE = apricot, RADIERER = eraser, KROKODIL = crocodile). The participant finally could read words with up to 14 letters (MUTTERINSTINKT = maternal instinct, PUPILLENREFLEX = pupillary reflex), a remarkable success according to her speech therapist who reported that she could not achieve comparable results with this patient during five weeks of rehabilitation.

When removing the cardboard assistance, some patients had difficulties in finding the target letters. So we instructed them to focus on the matrix and point to every letter they wished to spell before starting the spelling session. This procedure became superfluous in free-spelling. However, the time in which the screen stayed static to locate the next target letter was increased from 1000 ms to 4000 ms for all patients and all sessions.

### Spelling Performance

In this section, we reported accuracies of all patients based on the classification of EEG signals from all 12 electrodes (see Tables 3A,B). For the reported accuracies (online) we distinguished the cardboard presentation (Table 3A) from the standard presentation mode (Table 3B). In Table 3A, reached performances when using a re-trained classifier (ReC, offline) not considering occipital electrodes were added. Performances improved over sessions when using the cardboard paradigm (Table 3A).

**Table 3A:** **Performances (Perf) reached in percent correct per participant and word using the cardboard presentation mode**.

Participant		A	C	D	E
	Word to spell	Perf (ReC)			
Session 1	RADIO	Impossible	20% (20%)	100% (100%)	60% (40%)
	FUCHS	Impossible	20% (20%)	100% (100%)	-
	BLUME	Impossible	-	100% (100%)	-
Session 2	RADIO	60% (60%)	60% (40%)	-	-
	FUCHS	100% (100%)	60% (40%)	-	-
	BLUME	100% (100%)	80% (60%)	-	-
Session 3	RADIO	100% (100%)	100% (50%)	-	-
	FUCHS	100% (100%)	-	-	-
	BLUME	100% (100%)	-	-	-
Session 4	RADIO	100% (100%)	-	-	-
	FUCHS	-	-	-	-
	BLUME	100% (100%)	-	-	

*Accuracies reached with the re-trained classifier (ReC) were added*.

**Table 3B:** **Performances (Perf) reached in percent correct per participant and word using the standard presentation in the copy-spelling or free-spelling mode**.

Participant		A	B	C	D
Mode	Word to spell	Perf (ReC)			
Session 1	RADIO	-	100%	-	-
	FUCHS	-	100%	-	-
	BLUME	-	-	-	-
Session 2	RADIO	-	100%	-	100%
	FUCHS	-	100%	-	100%
	BLUME	-	100%	-	100%
Session 3	RADIO	-	-	-	100%
	FUCHS	-	100%	20%	100%
	BLUME	-	-	40%	100%
	TABELLEN	-	87.5%	-	-
FS	ICH	-	100%	-	-
FS	DORIS	-	-	40%	-
FS	INGE	-	-	-	100%
Session 4	RADIO	40%	-	-	-
	FUCHS	-	-	-	-
	BLUME	-	-	-	-
Session 5	RADIO	100%	-	-	-
	FUCHS	100%	-	-	-
	ANJA	100%	-	-	-
FS	HAUS	50%	-	-	-
Session 6	TAUBE	20%	-	-	-
FS	BUCH	100%	-	-	-
FS	ANJA	100%	-	-	-

*In case free-spelling mode was used, words were labeled (FS)*.

After participants had reached reliable performance of 100% using the cardboard presentation, the cardboard was removed and the participant used the standard BCI presentation in the copy-spelling mode and/or free-spelling mode (see Table 3B). Some participants chose the words to be spelled by themselves (see Table 3B) instead of sticking to the selection we offered. Participants A, B and D spelled freely. Participant E dropped out after the first session and therefore, we could not observe a potential training effect.

### Attention as a Predictor for BCI Performance

We hypothesized attention as measured using the TAP to predict spelling success with the BCI system. This hypothesis must be rejected. The attention indices as measured in the sample presented here were mostly far below average (see Table 4). However, patients were able to successfully use the BCI system. The TAP flexibility test could not be assessed in three patients (A, D and E) because instructions were not understood.

**Table 4:** **Results of the TAP subtests as indicated percentile rank (PR) and raw value (RV) for all participants (A–E)**.

Test		A	B	C	D	E
Alertness	RT (PR)	62	8	<1	3	<1
No signal	RT (RV)	215	332	643	444	811
Signal	RT (PR)	42	4	<1	5	<1
	RT (RV)	219	347	611	354	576
Divided attention	Errors (PR)	7	1	<1	<1	1
	Errors (RV)	5	22	61	78	26
	Omissions (PR)	3	<1	1	<1	1
	Omissions (RV)	6	14	9	50	11
Go/NoGo	RT (PR)	12	12	<1	96	1
	RT (RV)	501	510	1451	372	644
	Errors (PR)	4	18	1	1	18
	Errors (RV)	6	3	34	10	3
Flexibility	RT (PR)	Impossible	2	<1	Impossible	Impossible
	RT (RV)		2199	3398		
	Errors (PR)		2	<1		
	Errors (RV)		21	34		

### P300 Amplitude

**Figure 2:**
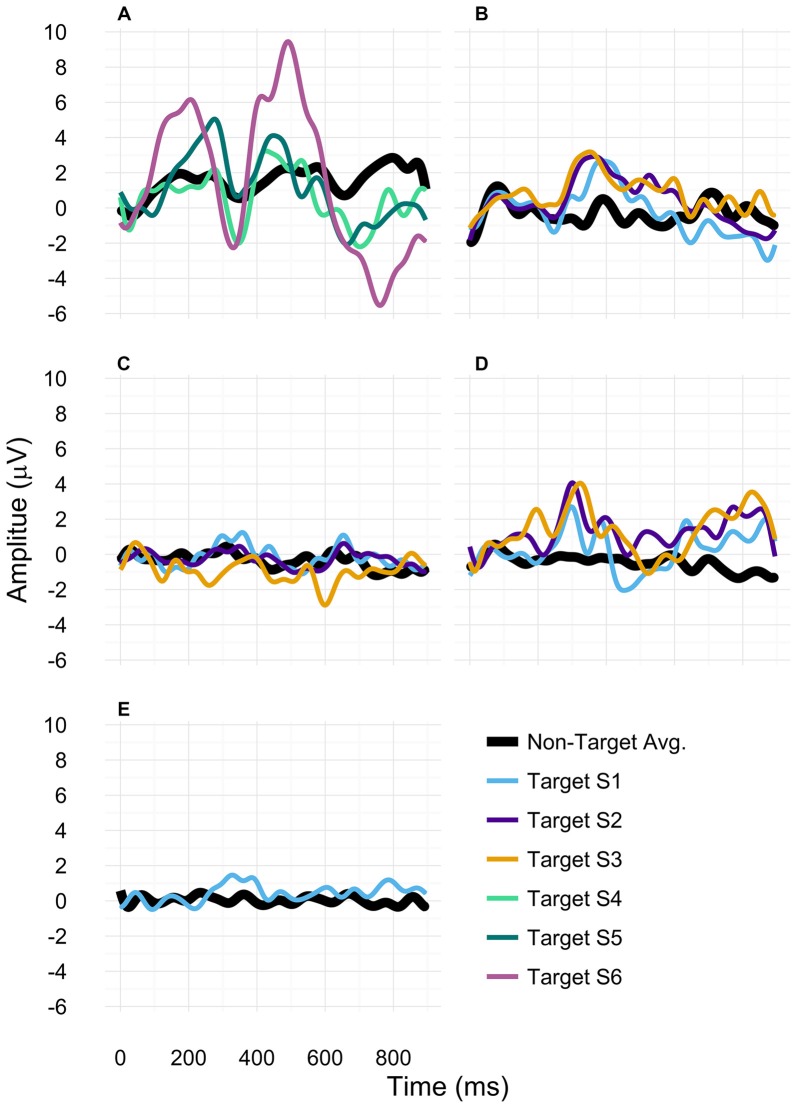
**P300 amplitudes for participants (A–E).** For participants **(A,B)**, only data assessed with the standard paradigm were included. For participant **(C)**, all depicted data were based on using the cardboard paradigm. For patient **(D)**, session 1 data was based on the cardboard paradigm and sessions 2 and 3 on the standard paradigm. For participant **(E)** only data assessed with the cardboard paradigm was displayed.

**Figure 3:**
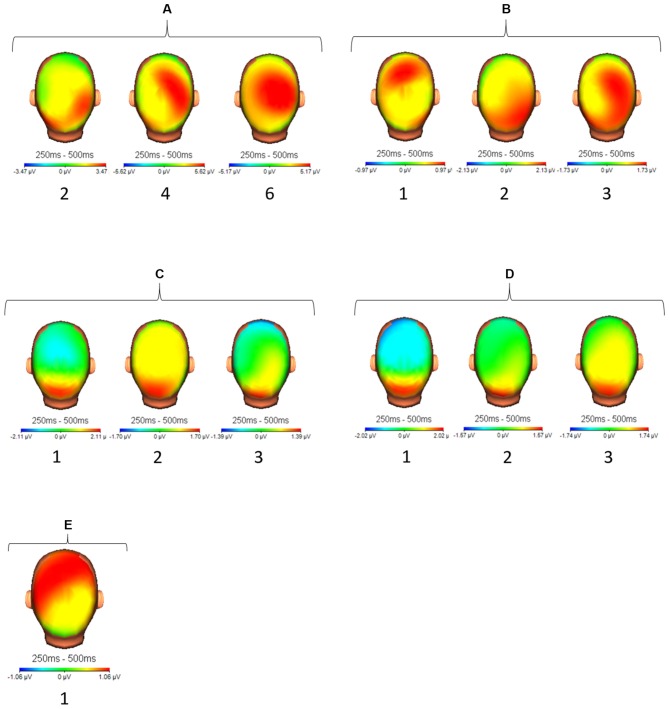
**Topographical display of activation (μV) between 250 ms and 500 ms after target stimulus presentation for all participants (A–E) and all sessions (1–3).** For participant **(A)** we selected sessions 2, 4 and 6 accordingly.

We hypothesized the P300 amplitude to increase with training. An increase of the P300 amplitude across presentation modes was found in patients A, B and D (see Figures 2, 3). In three patients we could remove the cardboard assistance and they finally spelled with high accuracies in the free-spelling mode (see “Spelling Performance” Section and Tables 3A,B). This suggests that participants could learn to produce classifiable EEG responses throughout the training which allowed for unassisted BCI use. However, as processes leading to ERP elicitation in the cardboard and the standard presentation might differ, we separated the reported P300 amplitudes for cardboard (Table 5A) and standard presentation mode (Table 5B).

**Table 5A:** **Average P300 amplitudes for each session and each participant at electrode position Cz using the cardboard (CB) presentation mode**.

Participant	A	C	D	E
μV				
Session 1	-	1.62	2.79	1.85
Session 2	3.73	1.11	-	-
Session 3	4.11	0.50	-	-
Session 4	5.71	-	-	-

**Table 5B:** **Average P300 amplitudes for each session and each participant at electrode position Cz using the standard presentation in copy-spelling mode**.

Participant	A	B	C	D
μV				
Session 1	-	2.76	-	-
Session 2	-	3.16	-	4.21
Session 3	-	3.50	1.97	4.22
Session 4	3.52	-	-	-
Session 5	4.31	-	-	-
Session 6	9.78	-	-	-

In patient A we found an increase of the P300 amplitude on Cz when comparing session 4 to session 6 of above 4 μV (standard presentation). In patient B, we found a slight increase of P300 amplitude from session 1 to session 2 and session 3 (standard presentation). In patient D, no P300 amplitude increase was found between sessions 2 and 3 (within standard presentation). In patient C, we found a decrease of P300 amplitude between session 1 and 2 and session 2 and 3 (cardboard presentation). As we measured patient E only once, we could not observe any changes in her P300 amplitude.

### Results of the Custom-Made Post-test Questionnaire

As patients could not read all given information or were unable to understand the questions, the experimenter supported the patients in answering the questionnaire. Questions to be answered on a VAS were read to patients. They were requested to indicate the position that represented their answer while the pen was moved from 0 to 10 by the experimenter. Patient E did not answer the questionnaire since she dropped out. The first question assessed subjective workload. Patients A and B indicated high subjective workload (8.2 and 9.8 out of 10) while patient C indicated medium workload (5.8) and patient D low workload (1.0). Concerning the BCI control strategy, three patients reported counting while patient D indicated that she had thought of the number 1 every time a target was highlighted. All participants were satisfied with the size of the letters. When being asked how easy it was to find the target letter, three patients reported difficulties and suggested the use of another color for the target stimulus or an otherwise more salient display (software solution). All patients reported that they could not think of a matrix array that would have supported them better in finding the letters in the matrix. Patients judged the cardboard cover as very helpful and could not think of another assistance that would have been more helpful to them. Patient A reported that he could not spell words containing more than six letters as the spelling procedure would be too time-consuming.

## Discussion

In this study, we investigated the potential use of a visual P300 BCI system in stroke patients diagnosed with aphasia. As our sample was very small and data could not be analyzed statistically, our results must be taken cautiously. Our results are not generalizable and represent first results of a feasibility study. However, we found that stroke patients diagnosed with mainly speech production deficits can successfully use a P300 based BCI after implementation of individualized adaptations and some training sessions. Nevertheless, it was challenging to assess the preliminary data presented here. All our participants were unable to communicate fluently and problems in understanding instructions were mainly expressed non-verbally. Even though this might seem trivial, special adaptation to the user’s needs were required as the experimenter had to consciously decelerate speaking rate (Liechty, 2006). Instructions needed to be repeated and participants had to be allowed time to express themselves. Orientation in the letter matrix had to be supported by naming the letters of the alphabet. Prior to data assessment, the experimenters started small talk conversation with each participant to deduce the ideosynchratic communication style (Neumann and Kübler, 2003). After participants showed consistent behaviors for a yes or no answer ranging from verbal communication to raising the hand or moving the head, the experiment was started. This procedure was successfully applied in all but one participant. As described in the “Materials and Methods” Section, he could not be included in the study because he neither could communicate consistently verbally nor non-verbally. The authors interpreted his body language and facial expressions such that he felt extremely uncomfortable with participation in the study and he could not give informed consent. However, we could not conclusively deduce what his thoughts were as also nurses who spent more time with him reported inconsistent communication patterns that could not be clearly interpreted. The multidisciplinary approach was absolutely crucial for this study (Kübler et al., 2014) as only by including recommendations of the medical doctors in charge and information by the caregivers we could cooperate successfully with the selected candidates.

Concerning the paradigm itself, adjustments to the existing protocols were necessary as we needed a cardboard cover to support the patients when they first tried to spell using the BCI device. Following the user-centered design (UCD) approach in BCI systems (Zickler et al., 2013; Kübler et al., 2014), we therefore increased usability and optimized effectiveness as with the standard paradigm, they could not have successfully spelled any letters. We judge the cardboard paradigm as a useful approach to familiarize the patient with the setup. This is also where its main benefit lies. The patient can be enabled to use the BCI in the free-spelling mode after training with the cardboard paradigm. However, saliency of a target stimulus might have been decreased by implementing the cardboard paradigm instead of highlighting the row and column in which the target letter was located. Future research should investigate possible effects of different adjustments to the paradigm proposed here. Concerning two other UCD aspects, satisfaction and efficiency, we reported possible changes to the protocol to improve satisfaction, such as facilitating attention allocation to the target character, but we did not aim at improved efficiency in terms of information transfer rates (Shannon and Weaver, 1964). Following the theoretical background we have presented here, the use of the BCI should support attention allocation (Hula and McNeil, 2008) and thereby boost cortical plasticity in language areas (Murray, 1999). Therefore, the use of the BCI system itself increases efficiency in terms of neuronal plasticity but not in terms of spelling speed, which in the approach presented here was not under investigation. With the cardboard paradigm, we cannot determine whether our participants only looked at or focused attention on the target letter. Their subjective reports in the custom-made questionnaire about their strategies for letter selection supported our assumption of deliberate attention allocation. However, future research needs to verify that the cardboard paradigm indeed facilitates attention allocation in patients with aphasia. We aimed at increasing attention and used the P300 amplitude as an indicator as it was shown that patients with motor aphasia usually still show a visual P300 (Knight and Scabini, 1998). Even though we did not assess changes in patients’ language abilities nor assessed a follow-up measurement using the TAP (Zimmermann and Fimm, 2012) as measurements would have been too close in succession to convincingly avoid training effects, we did find this increase of P300 amplitude in two patients. Inclusion of a higher number of training sessions would have allowed for multiple P300 amplitude comparisons within the same presentation mode (cardboard vs. standard), an aspect to be addressed by future research. Nolfe et al. (2006) measured the P300 in 17 patients with global aphasia in an auditory oddball paradigm and found those with the highest P300 amplitudes also showing best language abilities as measured with the Aachener Aphasia test (Huber et al., 1983). On the other hand, our TAP results clearly indicated attention deficits in all patients as they presented with very low PR in all measured categories, sometimes even results below PR1. TAP results definitely should be monitored in future studies, however, the assessed attention deficits here were not severe enough not to allow for BCI system use, which is a surprising result. Riccio et al. (2013) reported selective attention to have an impact on spelling accuracy in the P300 paradigm in their sample of patients with severe motor impairment. Our results clearly show BCI use being possible even if severe attention deficits do exist. Stroke patients who are diagnosed with aphasia might most likely also have additional diagnoses and it seems hard to find a homogenous sample. We included patients who differed in diagnoses and symptoms and also who presented some diagnoses which usually would be considered exclusion criteria, such as hemianopsia. Four of our patients were unable to use the P300 speller without the cardboard assistance in the first session. Against all odds, all patients learnt BCI use and spelled successfully in the free-spelling mode without assistance which supports the UCD idea of fitting the paradigm to the end-users’ needs.

It has already been reported in the literature that even chronic stroke patients (up to 11 years after stroke) can benefit from aphasia rehabilitation treatment and improve aphasia symptoms when given the opportunity (Basso et al., 1979). Intense and individualized speech rehabilitation were recommended as potentially successful even years after aphasia diagnosis. These findings were supported by reports on the successful use of constrained induced aphasia therapy (Meinzer et al., 2005). In their work, the authors had their participants perform 30 h of speech training within 10 days and reported significant improvement in a sample of 15 chronic stroke patients. Plastic reorganizational processes were hypothesized as one major cause of improvement. The authors judge personal involvement of patients by offering personally meaningful tasks as one potential factor contributing to success. However, stroke severity (Pedersen et al., 2003) and cognitive abilities at rehabilitation admission (Heruti et al., 2002) were identified as possible predictor variables for rehabilitation outcome. On the other hand, even though patients with a lower cognitive status were reported to benefit less from rehabilitation as compared to individuals with higher cognitive abilities, they also did benefit (Heruti et al., 2002). This finding supports the idea of offering rehabilitation also to patients who are diagnosed with mild cognitive impairments. For the paradigm presented here, cognitive functioning in terms of understanding instructions is a prerequisite. Therefore, participants who are unable to follow instructions must be rejected from the presented approach.

Further research is required to investigate the research idea presented here. Possible relations between attention, aphasia, visual P300 BCI use and rehabilitation success need to be investigated to evaluate the considerations presented here. To do so, a solid methodological design and training plan with a larger sample must be implemented as the presented study only confirms the feasibility of the suggested approach. If valuable, many stroke patients who suffer from aphasia and potential emotional disturbances, could be supported. Kauhanen et al. (2000) reported 70% of stroke patients with aphasia to fulfill the criteria for depression 3 months after stroke as compared to 46% of patients without aphasia. Thus, aphasia rehabilitation should become a focus of further research in rehabilitation, BCI research and psychology.

## Conclusion

The approach presented here needs further elaboration by future research. Paradigm adjustments to user requirements should be investigated and it is necessary to train bigger samples of participants using a thorough methodical approach that allows for statistical analysis and comparison with an adequate control group. However, preliminary data are promising and P300 based visual BCI use could be trained in participants who were unable to use the paradigm without individual adjustments.

## Author Contributions

SCK had the research idea, developed the concept and study design. Data analysis and manuscript writing were also performed by SCK. LG participated in the development of the study design and organized data acquisition. She measured EEG data and evaluated questionnaire results. ET and FXW were involved in patient selection and adjustments to the first version of the protocol following the user-centered design approach. Both gave advice for future studies and sample selection.

## Funding

This work was funded by the University of Würzburg, Institute for Psychology, Intervention Psychology.

## Conflict of Interest Statement

The authors declare that the research was conducted in the absence of any commercial or financial relationships that could be construed as a potential conflict of interest.
